# The use of porcine small intestinal submucosa mesh (SURGISIS) as a pelvic sling in a man and a woman with previous pelvic surgery: two case reports

**DOI:** 10.1186/1752-1947-3-70

**Published:** 2009-02-23

**Authors:** Osama Al-Sahaf, Sherif El-Masry

**Affiliations:** 1 Department of General Surgery, Our Lady of Lourdes Hospital, Drogheda, Co. Louth, Ireland

## Abstract

**Introduction:**

Closing the pelvic peritoneum to prevent the small bowel dropping into the pelvis after surgery for locally recurrent rectal cancer is important to prevent adhesions deep in the pelvis or complications of adjuvant radiotherapy. Achieving this could be difficult because sufficient native tissue is unavailable; we report on the use of small intestine submucosa extra-cellular matrix mesh in the obliteration of the pelvic brim.

**Case presentation:**

We describe two cases in which submucosa extra-cellular matrix mesh was used to obliterate the pelvic brim following resection of a recurrent rectal tumour; the first patient, a 78-year-old Caucasian man, presented with small bowel obstruction caused by adhesions to a recurrent rectal tumour. The second patient, an 84-year-old Caucasian woman, presented with vaginal discharge caused by an entero-vaginal fistula due to a recurrent rectal tumour.

**Conclusion:**

We report on the use of submucosa extra-cellular matrix mesh as a pelvic sling in cases where primary closure of the pelvic peritoneum is unfeasible. Its use had no infective complications and added minimal morbidity to the postoperative period. This is an original case report that would be of interest to general and colorectal surgeons.

## Introduction

Closing the pelvic peritoneum to prevent the small bowel falling into the pelvis following surgery for local recurrence of rectal cancer can be difficult because sufficient native tissue is unavailable. Small intestinal submucosa extra-cellular matrix mesh (SURGISIS; Cook Surgical, Bloomington, Indiana, USA) has been used to repair hernias, but it has never been documented to be used for pelvic inlet closure.

## Case presentation

### Case 1

Our patient was a 78-year-old Caucasian man with a past surgical history of Hartman's procedure performed in February 2005 for a perforated recto-sigmoid tumour. Postoperatively he developed pulmonary embolism and partial abdominal wound dehiscence. The fascial dehiscence was treated with daily dressings followed by elective closure three months later. The patient was not offered adjuvant chemotherapy because of his poor general health. He was followed up as an outpatient; this included a normal carcinoembryonic antigen (CEA) level, colonoscopy through the stoma, and computed tomography (CT) scan of the abdomen and pelvis. In October 2006 he presented to the accident and emergency (A&E) department with a three-day history of generalized colicky abdominal pain associated with vomiting, constipation and abdominal distension.

Examination showed tachycardia and dehydration. The abdomen was distended but soft with tenderness generally on deep palpation. There were exaggerated bowel sounds and digital examination through the colostomy showed no faecal impaction.

Routine bloods were normal except a raised C-reactive protein (CRP) of 92.9 and urea of 11.5. Plain film of the abdomen (PFA) showed dilated loops of the small bowel. CT scan of the abdomen and pelvis demonstrated a solitary liver metastasis in the right lobe of the liver, dilated loops of small bowel, and a soft tissue mass in the region of the rectal stump suspicious of a local recurrence. Conservative treatment was tried initially to relieve the obstruction, but the patient's condition did not improve. An exploratory laparotomy was then performed and the loop of small bowel adherent to the recurrent rectal tumour was mobilised and resected. The tumour itself was unresectable. To prevent the small bowel from falling into the pelvis, SURGISIS mesh was sutured to the sacral promontory, lateral pelvic wall and symphysis pubis to close the pelvic brim, and a tube drain was left in the pelvic cavity. The patient recovered well from the surgery with no complications. Neither adjuvant chemotherapy nor radiotherapy was advised because of his frail general condition and he has been followed up by both the surgical and palliative care teams as an outpatient.

### Case 2

Our patient was an 84-year-old Caucasian woman with a past surgical history of Hartman's procedure performed in 1993 for perforated sigmoid diverticulitis. The colostomy was reversed one year later. In 2005 she was diagnosed with a rectal tumour 10 cm from the anal verge. An anterior resection/Hartman's procedure was performed in May 2005. The tumour stage was Duke's B, but because of her age she was not considered for adjuvant therapy. In October 2006 she presented with per vaginal discharge of small intestinal contents.

CT of the thorax, abdomen and pelvis showed pulmonary metastases and local recurrence at the rectal stump with an entero-vaginal fistula. An exploratory laparotomy was performed, resecting the recurrent rectal tumour and the involved segment of small intestine; continuity of the small bowel was restored with a side-to-side anastomosis. The pelvic brim was closed with SURGISIS mesh sutured as in the first case to the sacral promontory, lateral pelvic wall and the symphysis pubis. Postoperatively the patient did well and was discharged home; she was later reviewed by an oncologist who opted against adjuvant therapy because of her age and poor general health. She is followed up by both surgical and palliative care teams as an outpatient.

## Discussion

A variety of abdominopelvic partitioning procedures designed to prevent the small bowel from coming into contact with the pelvic floor or the presacral area are described in the literature [1]. These procedures were introduced to prevent or ameliorate radiation-induced bowel injury associated with pelvic radiotherapy. A common technique is to create an omental sling and pack the pelvic space [2,3]. A significant drawback of this technique is that the omentum is frequently not large enough and may not possess the necessary tensile strength to adequately support the bowel loops. Thus the use of mesh was introduced. The two common types of absorbable mesh slings are Polyglycolic Acid (Dexon; Davis & Geck Co, Danbury, Connecticut, USA) or Polyglactin 910 (Vicryl; Ethicon Inc, Sommerville, New Jersey, USA), however both dissolve completely within 90 to120 days and the small bowel may ultimately fall back into the pelvis [4-6].

In our two cases, the pelvic peritoneum needed to be closed to exclude the small bowel from the recurrent tumour at the rectal stump and to facilitate postoperative radiotherapy. Because of previous pelvic surgery this was unfeasible and for that reason we used a prosthetic mesh that will encourage the growth of host tissues to prevent the small bowel from falling into the pelvis long after the mesh has dissolved. SURGISIS mesh is a three dimensional, acellular extra-cellular matrix comprised of collagen, noncollagenous proteins and other biomolecules including glycosaminoglycans, proteoglycans and glycoproteins harvested from porcine small intestine submucosa and made biocompatible using a patented process. It acts by providing a matrix for host connective and epithelial tissue growth and differentiation, resulting in a phenomenon called smart tissue remodelling as the implant and host tissue become indistinguishable [7]. Experimental evidence suggests that the tissue remodelling that replaces the matrix is stronger than the native tissue [8,9]. Additionally, the lack of permanent foreign material at the SURGISIS implant site [10] and rapid capillary penetration of the small intestinal submucosa and delivery of body defences to the local site may decrease the risk of mesh infection [11].

There is evidence that SURGISIS has been used successfully to repair congenital diaphragmatic hernias [12] and it has been used around the cardia to reinforce the gastrojejunal anastomosis during Roux-en-Y gastric bypass operations for morbid obesity, without complications [13]. Therefore, the literature suggests SURGISIS is safe around delicate organs and strong enough to prevent herniation of viscera through fascial defects.

The technique we used to fix the mesh was similar to that described by Devereux et al [14] with minor modification. After packing the small bowel into the upper abdomen the mesh was sutured to the retro-peritoneum at the sacral promontory, laterally to the lateral abdominal wall, and superiorly to the posterior sheath of the anterior abdominal wall above the symphysis pubis. Care was taken not to injure or entrap the ureters or iliac vessels. Closed suction drains were placed deep to the mesh to prevent collections because of the dead space created in the pelvis after elevating the small bowel. The placement of the mesh took approximately 40 minutes, which is similar to what is reported in the literature [15]. Both patients had mild postoperative ileus and a liquid diet was started on the sixth postoperative day. There were no mechanical complications in the early postoperative period nor pelvic abscesses or infections that could be related to the SURGISIS mesh.

## Conclusion

In cases where primary closure of the pelvic peritoneum is not feasible, the use of SURGISIS as a sling to hold the small bowel out of the pelvis can be performed without infective complications and adds minimum morbidity during the postoperative period. The use of SURGISIS as a pelvic sling has not been reported before and we suggest that future studies should compare SURGISIS slings to other abdominopelvic partitioning procedures.

## Abbreviations

SURGISIS: small intestinal submucosa extra-cellular matrix mesh; CEA: carcinoembryonic antigen; CT: computed tomography; A&E: accident and emergency; CRP: C-reactive protein; PFA: plain, film abdomen.

## Consent

Written informed consent was obtained from both patients for publication of this case report. A copy of the written consent is available for review by the Editor-in-Chief of this journal.

## Competing interests

The authors declare that they have no competing interests.

## Authors' contributions

OA reviewed patient charts and wrote the manuscript. SE critically reviewed the manuscript. Both authors read and approved the final manuscript.
